# *X* implies *Y* – Testing Hypotheses of Direction of Effect Using Configural Frequency Analysis

**DOI:** 10.1007/s12124-026-10011-6

**Published:** 2026-06-20

**Authors:** Alexander von Eye, Wolfgang Wiedermann

**Affiliations:** 1 Michigan State University, East Lansing, MI USA; 2 University of Missouri, Columbia, MO USA

**Keywords:** Configural Frequency Analysis (CFA), Statement calculus, Relevance logic, Conjunction, Implication, Confirmatory CFA testing

## Abstract

This article proposes using formal theory to specify models for confirmatory Configural Frequency Analysis (CFA). Statement calculus is used from the perspective of relevance logic. Under this perspective, patterns are identified that are ‘true’, that is, that are conform with a priori made statements. Among the true patterns, there also are patterns that reflect true statements although the premise is false – the well known ‘ex falso sequitur quodlibet’. These patterns are considered irrelevant, because there is no substantive connection between premise and outcome. This distinction has major implications for the selection of cells to be tested in confirmatory CFA: irrelevant cells are not considered as in support of a hypothesis. This is discussed for the operators conjunction and implication. For CFA, this approach requires an additional step. After performing standard CFA, tests are performed that examine the cells that support a hypothesis and the cells that are in contradiction to the hypothesis or irrelevant. If a hypothesis is confirmed, the cells supporting it contain more cases than expected under the CFA base model. Empirical data examples are given with data from a study on the effects of intimate partner violence.

Notoriously, measures that are based on second central moments, that is, covariances or correlations, are symmetric. That is, the order of variables can be interchanged without any effect on the resulting score. For example, for the correlation of metric variables, it holds that * cor(X,Y)=cor(Y,X)*, and, for covariances, it holds that $\:cov\left(X,Y\right)=cov(Y,X)$ (see Dodge, & Rousson, 2001; von Eye, & DeShon, 2012; Wiedermann, & von Eye, 2025). Similarly, for categorical variables, interactions or associations are symmetric as well. This can be illustrated as follows.

Consider the two binary variables *X* and *Y*. To represent the *X* × *Y* interaction, one performs the element-wise multiplication of the main effect terms of *X* and *Y*. Similarly, to represent the *Y* × *X* interaction, one performs the element-wise multiplication of the main effect terms of *Y* and *X*. As is well known, changing the order of multiplicands has no effect on the result of a multiplication. This is illustrated in Table 1.

**Table 1:** Representation of the *X* × *Y* and the *Y* × *X* interactions (main effects and interactions given in effect coding)

*X*	*Y*	Main Effect *X*	Main Effect *Y*	Interaction *X* × *Y*	Interaction *Y* × *X*
1	1	1	1	1	1
1	2	1	–1	–1	–1
2	1	–1	1	–1	–1
2	2	–1	–1	1	1

Evidently, the last two columns of Table 1 are the same. This can be viewed as parallel to the calculation of the cross-product to obtain the covariance of two variables, correlations, or regression coefficients. There is one consequence of the symmetry property of second central moments, namely, *using measures that are based on second central moments*,* hypotheses cannot be tested that involve direction of effect*.

A number of approaches has been proposed to solve this problem. Here, we briefly review four of these (for more detail, see, e.g., Wiedermann, & von Eye, 2016; Wiedermann et al., 2021), and resume the development of a fifth. The first approach is experimental research (Shadish et al., 2002). Experimental designs to create data do allow one to test directional hypotheses. This is clearly the preferred approach. However, not all research questions can be translated into experimental designs. For instance, research questions concerning the causes of diseases are, for ethical reasons, not amenable to experimental research, because they would require designs in which participants are made ill.

In a second approach, methods of statistical causal learning, such as *Linear Non-Gaussian Acyclic Models* (LiNGAM; cf. Shimizu et al., 2006, 2011) and *Direction Dependence Analysis* (DDA; see Wiedermann et al., 2021; Wiedermann, & Shi, 2027; Wiedermann, & von Eye, 2025) can be used to evaluate the direction of effects. These methods utilize third and fourth higher moments which conserve information that can be used for the analysis of direction of causation. These methods have been thoroughly developed, software exists (see, e.g., Ikeuchi et al., 2023; Wiedermann & Li, 2018; Wiedermann & Hirni, 2025), and a large number of application examples demonstrate their usefulness (see, e.g., García-Velázquez et al., 2020; Rosenström et al., 2012; Slaten et al., 2025; Wiedermann et al., 2020). In the present context, however, we need a different approach. LiNGAM and DDA were predominantly developed for continuous variables (for exceptions see Inazumi et al. 2011; Wiedermann and von Eye 2020a). Here, we focus on categorical variables.

A third approach was proposed by von Eye and Wiedermann (2021a, b). In this approach, variables or particular effect vectors are removed from a model, e.g., a log-linear model. When, after removal, other effects become non-significant, they are considered dependent on or caused by the removed terms. This approach, while applicable in both variable- and person-oriented research, is not pursued in this article either, because it does not allow one to test hypotheses about specific directed effects.

The fourth approach to discern statements of causal effect directionality focuses on the estimation of reciprocal effects, that is, cases in which *X* causes *Y* and vice versa (i.e., *X* ⇆ *Y*). Here, the predominant direction of effect can be established through comparing the magnitude and significance status of the *X* → *Y* and *Y* → *X* effects. For example, when the path coefficient of *X* → *Y* significantly deviates from zero and, at the same time, the corresponding reverse path *Y* → *X* does not reach statistical significance, one has found evidence that *X* is more likely to imply *Y*. Methods for the estimation of reciprocal effects often involve the use of instrumental variables (e.g., James & Singh, 1978). However, reciprocal effects can also be estimated without the presence of instrumental variables through incorporating higher moment information (cf. Ozaki & Ando, 2009). In the context of categorical variables Wiedermann and von Eye (2020b) proposed event-based reciprocal log-linear models which utilize Schuster-transformed non-orthogonal design matrices to estimate the intended *X* → *Y* and *Y* → *X* effects.

In this article, we continue the development of a fifth approach. This approach was first proposed by von Eye et al. (2026) in the context of a discussion of hypotheses that can be tested with Configural Frequency Analysis (CFA; Lienert 1968; von Eye and Gutiérrez Peña 2004; von Eye and Wiedermann 2021a, b*). *The hypotheses that can be tested are new to CFA. In all CFA models - they are called *base models* - hypotheses are represented that involve the entire cross-classification of the categorical variables that are examined. The new approach allows researchers to specify hypotheses that involve a selection of cells only.

The remainder of this article is structured as follows: First, we briefly review the new approach to testing directional hypotheses that concern selections of cells. Second, we extend this approach to cover more complex hypotheses, specifically, hypotheses of interest in longitudinal research and hypotheses of direction of effects. Real-world data examples will be given.

##  CFA of Selections of Cells

The CFA base model is specified using a falsificationism approach (Popper, 1959). Popper posits that hypotheses must be falsifiable to be considered scientific. There is a number of issues with this approach, for example the distinction between induction and deduction of hypotheses and theories. The discussion of theses issues is not pursued here (for an in-depth discussion, see, e.g., Lakatos, 1970). However, falsificationism is of use in CFA mainly for the following reason. To specify a CFA base model, hypotheses are split into two groups. The first contains the hypotheses that are *not* of interest to the researcher. The second group contains the hypotheses that are of interest. When the model that only includes the hypotheses from the first group is rejected, hypotheses from the second group, that is, hypotheses of interest, are bound to exist.

Virtually all existing base models of CFA can be specified using the following model (von Eye, & Wiedermann, 2025; von Eye et al., 2026)

$$\:\log\widehat m=\left[1\;X_{G}\left|\;X_{A}\left|\;X_{B}\right|X_{\dots}\right|\;X_{C}\right]\;\begin{bmatrix}\lambda_{c}\\\Lambda_{G}\\\Lambda_A\\\Lambda_{B}\\\Lambda_{\dots}\\\Lambda_C\end{bmatrix}$$

where

$\:\widehat{m}$ : is the vector of estimated cell frequencies,
1: is the constant vector,
*X*_*G*_: is that part of the design matrix that contains the vectors for the effects that distinguish groups of data carriers,
*X*_*A*_: is that part of the design matrix that contains the vectors for the main effects and interactions in Group A,
*X*_*B*_: is that part of the design matrix that contains the vectors for the main effects and interactions in Group B,
*X*_…_: are those parts of the design matrix that contain the vectors for the main effects and interactions in additional groups,
*X*_*C*_: is that part of the design matrix that contains vectors for covariates,
λ_*c*_: is the parameter of the model constant,
Λ_*G*_: are the parameters of the grouping variables,
Λ_*A*_: are the parameters of the main effects and interactions in Group A,
Λ_*B*_: are the parameters of the main effects and interactions in Group B,
Λ_…_: are the parameters of the main effects and interactions in additional groups, and
Λ_*C*_: are the parameters of the covariates,

The vertical bars in the above equation carry no operational function. These were inserted just to separate the vectors in the design matrix.

 There are two ways to use such a CFA base model. The first is exploratory. The entire cross-classification is searched for cells (configurations) that contain significantly more (or fewer) cases than expected from the base model. These cells are said to constitute CFA types (or antitypes). The second is confirmatory. Individual cells or groups of cells are examined. These cells are hypothesized to represent particular effects.

In the models discussed in the following sections of this article, these effects are not main effects or interactions from a hierarchical or non-hierarchical log-linear model. Instead, these are cells that represent hypotheses that cannot be represented by hierarchical models. These hypotheses are, therefore, represented in non-standard models (see Mair, & von Eye, 2007). Some of these hypotheses can be included in the base model given above. They would be included in the form of covariates, in the vectors denoted by *X*_*C*_. Other hypotheses result in the selection of cells to be examined, in addition to the model given above. These are the hypotheses to be discussed in this article. In the following section, we describe, how these hypotheses can be derived. For this purpose, we use statement calculus.

## Statement Calculus and CFA Hypotheses

Statement calculus (propositional logic) processes statements (propositions) and connections between them (Bauer, & Wirsing, 1991; Franks, 2023). It does not process non-logical objects, that is, the semantic content of statements. It also does not process quantifiers. It solely is used to determine whether statements are, formally, *true* or *false* (two-valued logic).

Before, however, describing how to incorporate statement calculus into CFA, the distinction between *formal* and *material elements* of statements needs to be made explicit (see, e.g., Beall, Restall. & Sagi, 2024). Statement calculus is abstract in the sense that it does not take into account features of objects that are examined. That is, a statement is either true or false *no matter the semantic content*, and it cannot be altered by empirical knowledge. In other words, the semantic content of a statement is not part of the decision concerning its truth. In addition, the rules of statement calculus do not add semantic content to statements. Instead, they show ways to combine semantic content and structure it. Thus, *formal elements* involve the rules of logic, and *material elements* are concerned with the semantic content. Statements are either true or false. Material consequences represent a priori knowledge, that is, knowledge that is not generated by experience (observations, experiments, recordings).

The role of CFA is to create *a posteriori* knowledge, that is, e.g., evidence from observations or experiments. In the context of statement calculus, and using the tools of statistics, CFA creates material knowledge. This knowledge can be processed following the rules of logic. More specifically, given a cross-classification of categorical variables, statement calculus is used to identify those cells that are conform with those patterns of a statement that are true, and those patterns that are false. This applies to elementary statements as well as to composite statements, that is, statements that result from linking one or more elementary statements.

In the following paragraph, we review how statement calculus can be used to identify true patterns of statements. We then relate true patterns to the search for types and antitypes in CFA (cf., von Eye, & Wiedermann, 2026). Table 2 displays the truth values for elementary statements and composite statements, that is, statements that link two elementary statements (cf., Boole’s, 1854, or Wittgenstein’s, 1905, truth tables), for four operators (see also Brandtstädter, 1991).

**Table 2:** Truth values {T, F} for the elementary statements *X* and *Y*, and the operators *and* (conjunction; ∧), *or* (disjunction; ∨), *implication*, (→), and *reverse implication* (←)

*X*	*Y*	*X* ∧ *Y*	*X* ∨ *Y*	*X* → *Y*	*X* ← *Y*
T	T	T	T	T	T
T	F	F	T	F	T
F	T	F	T	T	F
F	F	F	F	T	T

In Table 2, the statements *X* and *Y* are either true or false. They are crossed, with the effect that each truth value of *X* co-occurs with each truth value of *Y*, and vice versa (Columns 1 and 2 in Table 2). The following columns show the truth values for composite statements that link *X* and *Y*. The column under the conjunction *X* ∧ *Y* shows that a conjunction is true only when both, *X* and *Y* are true. A disjunction, shown in the column under *X* ∨ *Y*, is true when one or both of *X* and *Y* are true. The implication, shown under *X* → *Y*, is true when both, *X* and *Y*, are true, or when *X* is false. This applies accordingly to the reverse implication, *X* ← *Y*. This composite statement is true when both, *X* and *Y* are true or when *Y* is false.

One might ask why an implication is true when the premise is false. There are several responses to this question. The best known of these is that *ex falso sequitur quodlibet*, that is, from something false, anything can follow. The second, linked to the first, is that the instances in which the premise is false result in *vacuously true* composite statements (cf. Elkind, 2021). That is, the composite statement is true, but irrelevant, because the premise is false.

The third answer is that, in *relevance logic*, there is a distinction between strict and material implication (cf. Beall, Restall. & Sagi, 2024; Mares, 2024). Here, also, a lack of relevance can be seen. This lack manifests when the conclusion has nothing to do with the premise. Consider the following example. When the restaurant reservation is confirmed in Montpellier, France, it rains in Columbia, Missouri. Formally, this implication can be true, but the premise and the conclusion have nothing to do with each other. In other words, the premise is not of relevance for the conclusion. Evidently, relevance logic moves away from processing the truth of statements solely on the basis of form. It also considers the material content of statements. The *ex falso sequitur quodlibet* no longer applies. Being concerned with the creation of material knowledge, we follow, in the present article, an approach with focus on statements that are formally true, material, and in which the premise is relevant for the conclusion.

As was stated in the first section of this article, confirmatory CFA hypotheses are formulated in a context of falsificationism. That is, the CFA base model represents the negation of the hypotheses of interest. In the present context, it can be said that the base model represents statements that are hypothesized to be either irrelevant or false. The base model must be specified such that the hypotheses of interest are represented by statements that are true. Consider, again, Table 2. If the hypothesis of interest posits *X* ∧ *Y*, the base model must be specified such that a link between *X* and *Y* is negated. This is the case when

1. *X* and *Y* are independent,
2. a type appears in the cell in which both, *X* and *Y*, are true, and
3. antitypes emerge in the cells in which the composite statement, *X* ∧ *Y*, is either irrelevant or false.

It is interesting to see that this specification represents a concept of association (or correlation) that differs from the standard concept. The standard concept focuses on the diagonal of a square cross-classification. If this concept is pursued, the cells in which only one of *X* and *Y* is true, that is {T, F} and {F, T}, are irrelevant, and a type is expected for Cell {T, T} and an antitype is expected for Cell {F, F}. In the two irrelevant cells, neither types nor antitypes are expected to emerge (for a discussion of the interpretation of correlations, see Rovine & von Eye, 1997). Here, Cell {F, F} is considered irrelevant as well because the premise was not observed.

Similarly, when the hypothesis of interest posits *X* ∨ *Y*, a base model must be specified that also negates a link between *X* and *Y*. This is the case when

1.  *X* and *Y* are independent,
2. there appear types in the cells in which either just one of *X* and *Y* is true, or both, and
3. there appears an antitype in the cell in which both, *X* and *Y*, are false.

When the hypothesis of interest posits that *X* implies *Y*, that is, *X* → *Y*, a base model must be specified that negates a link between *X* and *Y* as well. The proposition can be considered confirmed when

1. *X* and *Y* are independent,
2. a type appears in the cell in which both, *X* and *Y*, are true, and
3. an antitype emerges in the cell in which *X* is true and *Y* is false.

The cells with truth values {T, F} and {F, T} are considered irrelevant. This applies accordingly for the reverse implication (see von Eye et al., 2026).

In the following sections, we present real-world data examples. In each of these, we illustrate confirmatory CFA of hypotheses that go conform with statements that are true in the context of relevance logic. In addition, we compare the results from this approach with results from standard exploratory CFA.

## Data Examples

In the first example, we resume the analysis of data from a project on the effects of intimate partner violence (see Bogat et al., 2004; Huth-Bocks, 2024; Bogat, Levendosky, & von Eye, 2004). In this project, 204 pregnant women responded, on four occasions, to questions concerning their experiences with violence that was perpetrated by their intimate partners. In the example, we use data from the first two occasions. We analyze the responses to intimate partner violence, experienced in the time periods before the interviews (coded as 1 = did not and 2 = did experience violence), and stability of mood, given on the second occasion (coded as 1 = stable and minimally variable and 2 = unstable).

The first base model that we apply is that of standard CFA of variable independence. The base model for this hypothesis is $\:\mathrm{log}\:\widehat{m}=\lambda\:+{\lambda}^{V1}+{\lambda}^{V2}+{\lambda}^{M},$ where *V*1 and *V*2 indicate Violence reported in the first two interviews, and *M* indicates Mood Stability, reported in the second interview. For this and the second analysis, we use the *z*-test and protect the nominal significance level using the Holland-Di Ponzio Copenhaver procedure. Table 3 displays the results for this analysis.

**Table 3:** Exploratory CFA of variable independence of Violence, reported in two interviews, and Mood Stability, reported in the second interview

*V*1 *V*2 *M*	*m*	*\widehat m*	*z*	*p*	
111	35.00	18.979	3.6773	0.000118	Type
112	40.00	32.290	1.3569	0.087413	
121	26.00	26.924	−0.1781	0.429305	
122	23.00	45.806	−3.3697	0.000376	Antitype
211	3.00	12.857	−2.7490	0.002989	Antitype
212	8.00	21.874	−2.9664	0.001506	Antitype
221	13.00	18.239	−1.2267	0.109959	
222	60.00	31.030	5.2006	0.000000	Type

Overall, the goodness-of-fit for the base model that includes all main effects is poor (LR $\:{\chi}^{2}$ = 71.99; *df* = 4; *p* < 0.001). The model is, therefore, rejected, and we can expect types and antitypes to emerge. There are two types and three antitypes. The types are constituted by Configurations 1 1 1 and 2 2 2. They suggest that when there is no violence over the entire observation period, mood remains stable (1 1 1), and that when there is violence over the entire observation period, mood remains unstable (2 2 2). The antitypes (1 2 2, 2 1 1, and 2 1 2) suggest that it is less likely than compatible with the model of variable independence that mood is unstable, when violence emerges (1 2 2). It is also less likely than compatible with the model of variable independence that mood becomes stable when violence decreases (2 1 1). The same applies for unstable mood (2 1 2). The last two antitypes can be assumed to reflect the effect that a decrease in violence is rare.

In the second CFA, we use a different base model. We take into account that there may be an association between the two responses to intimate partner violence. If this association exists, types and antitypes in Table 3 possibly reflect this association instead the association between the two violence responses and mood (in)stability. The base model for this CFA is $\:\mathrm{log}\:\widehat{m}=\lambda\:+{\lambda}^{V1}+{\lambda}^{V2}+{\lambda}^{V1,V2}+{\lambda}^{M},$ where the doubly superscripted term represents the association between *V*1 and *V*2. In other contexts, this model has been termed the model of Prediction CFA (P-CFA; Heilmann et al., 1979; cf., von Eye and Wiedermann 2021a, b). Table 4 presents the results of P-CFA.

**Table 4:** Exploratory Prediction CFA of Violence on two occasions and Mood Stability on the second occasion

*V*1 *V*2 *M*	*m*	*\widehat m*	*z*	*p*	
111	35.00	27.764	1.3732	0.084848	
112	40.00	47.236	−1.0528	0.146220	
121	26.00	18.139	1.8456	0.032474	
122	23.00	30.861	−1.4150	0.078536	
211	3.00	4.072	−0.5313	0.297609	
212	8.00	6.928	0.4073	0.341885	
221	13.00	27.024	−2.6977	0.003491	Antitype
222	60.00	45.976	2.0683	0.019307	

Overall, the goodness-of-fit for the base model that includes all main effects and the two-way interaction between the two Violence responses is poor as well (LR $\:{\chi}^{2}$ = 21.50; *df* = 3; *p* < 0.001). However, the model is significantly better than the model of variable independence (Δχ^2^ = 50.46; Δ*df* = 1; *p* < 0.001). Clearly, the association between the two violence responses explains a significant portion of the variability in this cross-classification. Still, the model is rejected, and we expect types and antitypes to emerge.

Table 6 shows that each type and antitype that emerged in the CFA of variable independence has disappeared. This is solely due to including the interaction between the two violence responses in the base model. The types and antitypes in Table 3 can, therefore, not be interpreted as reflecting the relation between the two violence responses on one side and Mood Stability on the other. Under the P-CFA base model, only one antitype emerges, and no type. The antitype suggests that it is less likely than expected under this base model that violence in both observation periods is linked to stable mood reported in the second interview.

For the interpretation of this antitype and for the following discussion, it is important to realize that, the prediction antitype in Table 3 does not allow one to conclude that a (causal) path goes from the two violence responses to stable mood. As was emphasized by Krauth (1993, p. 138), “…* an association does not indicate direction of effect from one configuration to an other. Also, associations can exist between variable groups that are not directly related.*” Therefore, if one intends to make statements that involve directed relations, a different approach is needed.

Applying the concepts described in the second section of this article, this approach proceeds in the following steps. First, a decision is made concerning the link between variables. To make this decision, operators are selected from statement calculus. When two variables are analyzed, one selects one operator. When more than two variables are analyzed, multiple operators are needed. It is important to realize that none of the links has a corresponding form in standard statistics. Consider, for instance, the conjunction, the logical *and*. In the present context, to study the conjunction of two variables, one focuses on the sole cell for which both variables are true. That is, one focuses on those cases for which values of the two variables were observed that correspond to *true* statements. For example, when diagnoses of ADHD are examined, one might focus on inattention and impulsivity. For the conjunction to be true, both must have been observed. The remaining cells of the cross-classification can be considered irrelevant. In contrast, when the disjunction is used, all cells are in focus in which at least one of the symptoms were observed. The only irrelevant cell is the one with cases for which none of the symptoms was observed.

When more than two variables are observed, multiple operators can be used. Consider, for example, the data in Tables 3 and 4. The first two variables, Violence reported in the first and the second interviews, were treated differently in the two CFAs. In the first, they were hypothesized to be independent. In the second CFA, they were hypothesized to be associated. The third variable, Mood Stability, was hypothesized to be independent of both violence variables. The results in Table 4 suggest that the two violence variables are significantly related, which invalidated the results of the first CFA.

In the analyses that follow, we link statement calculus with CFA. We first select one or multiple operators to link *V*1, *V*2, and *M* and, then, in the second step, we specify a CFA base model. This model is constructed according to the same rules as standard CFA base models. Specifically, variables that are hypothesized to be related are, following the falsification approach, specified to be independent. In the third step of the analysis, we perform CFA tests on the individual cells that represent true statements. Irrelevant cells are tested using tests for groups of cells. Specifically, we perform Stouffer’s *Z* test (Stouffer et al., 1949),

$$\:Z=\frac{\sum_{j=1}^{J}{z}_{j}}{\sqrt{J}}$$

where the *z* scores represent the significance tests of the cells of interest, under a particular CFA base model. This test is a probability pooler. That is, it synthesizes the probability of multiple events. The test calculates the average of the *z* scores of the cells that are examined. In sum, we perform a confirmatory CFA.

The focus on cells that represent true statements alters the procedure of α protection. In exploratory CFA, each of the *t* cells of a cross-classification cell is routinely subjected to a CFA test. The first protected α is, therefore, α* = α/*t* (for special cases and exceptions, see von Eye and Wiedermann 2021a, b). Here, the magnitude of the first protected α depends on the number of true cells that are not considered irrelevant. When this number is *t*_*i*_, the first protected α becomes α* = α/*t*_*i*_, with *t*_*i*_ < *t*. One-sided testing can be considered. When there is only one cell that is ‘true’ and relevant, the protected α equals the nominal α.

### CFA and Relevance Logic: Data Examples

In this section, we resume the configural analysis of the data in Tables 3 and 4. Here, we perform confirmatory CFA. We derive from relevance logic, which cells are subjected to which form of CFA testing. We conduct three CFAs. In the first, we posit that the three variables, *V*1, *V*2, and *M* are connected by way of conjunction, or *V*1 ∧ *V*2 ∧ *M*. In the second, we posit the double implication *V*1 → *V*2 → *M*, that is, we posit that violence reported at the first interview implies violence reported at the second interview, and that the latter implies Mood Instability, reported also at the second interview. In the third CFA, we posit that (*V*1 → *V*2) ∧ (*M* → *V*2) or, in words, that violence reported at the first interview implies violence reported at the second interview, which stands in conjunction with the implication of violence reported at the second interview from Mood Instability. The three variables, *V*1, *V*2, and *M* are materially connected in the sense that they are observed twice (*V*1 and *V*2) or in the same context, that is, intimate partner violence. Therefore, they can be treated from the perspective of relevance logic.

In the first run, we posit that the three variables, *V*1, *V*2, and *M* are connected in the sense of a logical *and*, that is, in the form of the double conjunction *V*1 ∧ *V*2 ∧ *M.* In words, we posit that violence reported in the first two interviews and unstable mood co-occur. The truth table for this hypothesis is given in Table 5.

**Table 5:** Truth table for the proposition *V*1 ∧ *V*2 ∧ *M*

*V1*	*V2*	*M*	*V1* ∧ *V2*	*V1* ∧ *V2* ∧ *M*
**T**	**T**	**T**	**T**	**T**
T	T	F	T	F
T	F	T	F	F
T	F	F	F	F
F	T	T	F	F
F	T	F	F	F
F	F	T	F	F
F	F	F	F	F

Table 5 shows that the sole configuration that supports the hypothesis is {T, T, T} (printed in bold, in the first line of the table). In the base model, the hypothesis is represented by the proposition of variable independence, or $\:\mathrm{log}\:\widehat{m}=\lambda\:+{\lambda}^{V1}+{\lambda}^{V2}+{\lambda}^{M}$ that had been used already for the first analysis of the intimate partner violence data, in Table 3. As was shown in the first analysis, the base model is rejected, and we can expect types and antitypes to emerge. In contrast to the first exploratory analysis, we now focus on just one cell of the *V*1 × *V*2 × *M* cross-classification. This is Cell 2 2 2. All other cells are either irrelevant, because either *V*1 or *V*2 are false, or both, or they contradict the proposition because the consequent (*M*) is false (Cell 2 2 1).

Table 3 shows that Configuration 2 2 2 does indeed constitute a CFA type. It, therefore, supports the hypothesis that the three variables are linked in the form of a conjunction. Cell 2 2 1 constitutes neither a type nor an antitype. It, therefore, supports the hypothesis of the base model, that is, the hypothesis that the three variables are independent.

To complete the analysis of the hypothesis of conjunction, we ask whether the irrelevant cells – here, they all contradict the hypothesis – jointly contain fewer cases than expected under the assumption of independence. In the present data example, we obtain, for all irrelevant cells, that is, all cells except 2 2 1 and 2 2 2, the Stouffer test statistic *Z*_{1 1 1, …, 2 1 2}_ = −1.726. The corresponding tail probability is *p* = 0.042 (one-sided testing). We conclude that the irrelevant configurations that contradict the hypothesis of conjunction jointly constitute an antitype, and that, in all, the hypothesis of a double conjunction can be retained.

In the second example of the link of CFA and relevance logic, we ask whether a hypothesis of directed effect can be retained (cf., von Eye et al., 2026). The hypothesis we test is one of double implication. Specifically, we posit that *V*1 → *V*2 → *M*. In words, we posit that Violence reported at the first interview implies violence reported at the second interview which, in turn, implies unstable mood, reported also at the second interview. The base model for this proposition is the same as for the proposition of conjunction, that is, the model of variable independence. The analyses summarized in Table 3 suggest rejecting this base model. We, therefore, can expect types and antitypes to emerge. The configuration that contains the cases for which the proposition of double implication holds is 2 2 2. This is the same as for the proposition of conjunction. Table 6 shows the truth values for the proposition of double implication.

**Table 6:** Truth table for the proposition *V*1 → *V*2 → *M*

*V* *1*	*V* *2*	*M*	*V1 →* *V2*	*V1 →* *V2 →* *M*
**T**	**T**	**T**	**T**	**T**
T	T	F	T	F
T	F	T	F	T
T	F	F	F	T
F	T	T	T	T
F	T	F	T	F
F	F	T	T	T
F	F	F	T	F

Evidently, based on the sole pattern that contains only ‘true’ values, the conjunction and the implication hypotheses cannot be distinguished (see the first rows in Tables 5 and 6). The same holds for the sole pattern that contains ‘true’ values for the violence responses and a ‘false’ value for the mood instability response. The differences between the double conjunction and the double implication hypotheses appear in the remaining seven patterns. In the conjunction hypotheses, they all are ‘false.’ In the implication hypotheses, four of the remaining patterns are ‘true’ and three are ‘false.’

As in the double conjunction example, we begin the statistical analysis of the double implication hypothesis with the rejection of the base model of variable independence. In addition, the critical cell, 2 2 2, again suggests retaining the double implication hypothesis^1^. This, however, is not sufficient. We need to find a way to distinguish the two hypotheses. In the analysis of the double conjunction hypothesis, we performed a Stouffer test for the remaining six cells. Each of these cells contained a ‘false’ value, but they were irrelevant because one or more of the truth values of the premise were ‘false.’ Here, we can distinguish between the three cells that contain ‘true’ and the four cells that contain ‘false’ values (see Table 6). Each of the four cells that contain ‘true’ values contains at least one response to *V*1 or *V*2 that is ‘false.’ These cells are irrelevant because the premise is not fulfilled, according to which *V*2 is implied by *V*1. For the three cells that contain ‘false’ values, each contains a ‘false’ value for stable mood. These cells are irrelevant because the implication that stable mood is implied is not fulfilled.

We perform separate Stouffer tests for these two groups of cells. For the four cells for which the composite double implication is ‘true,’ that is, for Cells 1 1 2, 1 2 2, 2 1 1, and 2 1 2, we obtain *Z* = −3.864, with *p* < 0.001. This result suggests that it is less likely than expected under the base model that responses suggest that the outcome is ‘true’ when the premise is not fulfilled. For the two cells for which the composite double implication is ‘false’, that is, for Cells 1 1 1, and 1 2 1, we obtain *Z* = 2.474, with *p* = 0.013. This result suggests that, in irrelevant cells, the occurrence of stable mood is, under the assumption of variable independence, more likely than expected.

In sum,

1. the rejection of the base model of variable independence,
2. the type that is constituted by Cell 2 2 2,
3. the rejection of the independence hypothesis for the irrelevant cells for which the composite implication is ‘true’ (rejected because the outcome ‘true’ is less likely than expected under the base model), and
4. the rejection of the independence hypothesis for the irrelevant cells for which the composite implication is ‘false’ (rejected because the outcome ‘false’ is more likely than expected under the base model),

can be interpreted as in support of the double implication hypothesis at the level of variable categories.

In the third CFA, we test a more complex hypothesis. Specifically, we test the hypothesis that *V*1 implies *V*2 and *M* implies *V*2. Table 7 presents the truth value for this composite hypothesis, including, for comparison, the hypotheses of the previous analysis, *V*1 → *V*2 → *M*.

**Table 7:** Truth table for the hypotheses *V*1 *→V*2* →M* and (*V*1*→V*2) ∧* (M→V*2*)*

*V* *1*	*V* *2*	*M*	*V1 →* *V2*	*V1 →* *V2 →* *M*	*M* *→ V2*	*(V1 →V2) *∧ *(M→V2)*
**T**	**T**	**T**	**T**	**T**	**T**	**T**
T	T	F	T	F	T	T
T	F	T	F	T	F	F
T	F	F	F	T	T	F
F	T	T	T	T	T	T
F	T	F	T	F	T	T
F	F	T	T	T	F	F
F	F	F	T	F	T	T

As in the previous two CFAs, Configuration 2 2 2 confirms the hypothesis. All involved variables are ‘true.’ Four additional cells are ‘true.’ However, they are irrelevant because either *V*1, *M*, or both are ‘false.’ The remaining three cells are ‘false’ and irrelevant because *V*2, the outcome is ‘false.’ This pattern differs from the one for double implication. The four ‘true’ cells 1 1 1, 1 2 1, 1 2 2, and 2 2 1 are irrelevant because either *V*1 or *M*, or both are ‘false.’ The three ‘false’ cells 1 1 2, 2 1 1, and 2 1 2 are irrelevant also because one or both of the premises are ‘false.’

For the group of ‘true’ cells, we calculate the Stouffer *Z* = −0.549, with *p* = 0.583. This suggests that, jointly, these cells do not significantly differ from the expectation that is based on the assumption of variable independence. For the group of ‘false’ cells, we calculate the Stouffer *Z* = −2.516, with *p* = 0.012. This suggests that, jointly, these cells represent significantly fewer responses than expected based on the assumption of variable independence.

In sum,

5. the rejection of the base model of variable independence,
6. the type that is constituted by Cell 2 2 2,
7. the retaining the independence hypothesis for the irrelevant cells for which the composite implication is ‘true,’ and
8. the rejection of the independence hypothesis for the irrelevant cells for which the composite implication is ‘false’ (rejected because the outcome ‘true’ is less likely than expected under the base model),

can be interpreted as in support of the hypothesis that, in this data set, two implications are active according to which *M* is implied by *V*1 *and* by *V*2.

## Discussion

The present article makes a contribution to the event-based analysis of hypotheses (cf. von Eye, & Wiedermann, 2017, 2018). The frequency of occurrence of events is analyzed at the level of variable categories instead of degree, intensity, or magnitude at the level of metric variables. A first benefit from this approach is that patterns of ‘did occur’ and ‘did not occur’ can be examined and modeled. A second benefit is that analyses can be performed not only at the level of variables as in log-linear modeling, but also at the level of individual configurations as performed in CFA in the context of person-oriented analyses.

The third and greatest benefit is that this approach allows researchers to empirically evaluate formal theory. Earlier attempts to perform such analysis did not use relevance logic. Instead, they proposed establishing *prediction logic* (e.g., Hildebrand et al., 1974, 1976). The advantage of the present approach is that it allows one to use results from statement calculus within the framework of relevance logic, and test hypotheses that are material, relevant, and compatible with formal theory.

In the present article, we first derived hypotheses that reflect such logical operators as conjunction and implication within the context of the effects of intimate partner violence. The configurations were identified that are in support of conjunction, implication, or both. Second, the configurations were identified that are in contradiction to conjunction or implication, and, third, the configurations were identified that are irrelevant for the hypotheses. This was possible because the ‘ex falso sequitur quodlibet’ does not apply in relevance logic (this issue is not discussed in detail in the context of Hildebrand et al.’s, 1974, 1976, prediction logic). Based on these configurations, the differences between conjunction and implication can be ascertained.

The use of categorical variables and relevance logic does allow one to empirically test statements that are formally true. The method of choice for these tests is CFA. It operates at the level of individual configurations or groups of configurations. The data examples given in this article illustrate both. Relevance logic requires that the propositions that are linked by, e.g., conjunctions or implications, share material elements. If this is not the case, they are irrelevant. In the examples given in this article, there clearly were shared elements. Whenever there is no such material connection, cells turn irrelevant and are moved into different groups of cells when probability poolers are employed.

An interesting and important element of the method proposed here is that the same configurations can stand out in different contexts. In Table 3, Configuration 2 2 2 constitutes a CFA type. The base model for this CFA (and the following ones) was the model of variable independence. When applied in exploratory CFA, this cell simply suggests that the hypothesis of variable independence is violated by Configuration 2 2 2 which was observed significantly more often than expected. The same configuration also stood out in the first empirical evaluation of formal theory. In this evaluation, a double conjunction was posited. Therefore, the interpretation of the type that was constituted by Configuration 2 2 2 is different. It no longer just contradicts a model that operates at the level of variable associations. Instead, it is one element of the confirmation of the hypothesis that the three variable categories co-occur. The second element is that all other cells, that is, the cells that contradict the hypothesis, jointly occur less often than expected under the base model.

It is important to note that Table 3 contains a second type. It is constituted by Configuration 1 1 1. In exploratory CFA, this type suggests that these three categories occur significantly more often than expected. In the analysis of the double conjunction, this configuration is part of those that, together, occur less often than expected. Evidently, the interpretation of frequencies as more versus less often than expected depends on the hypotheses that are being tested.

This applies accordingly to the analysis of the double implication hypothesis. This hypothesis has been discussed in the contexts of directed hypotheses and causal analysis (Bogat et al., 2021; von Eye et al., 2026). Here, we illustrate one additional feature. This is the feature that cells are irrelevant only with respect to an implication. They are not irrelevant at all when it comes to the distinction between hypotheses. Here, they are needed to distinguish between conjunctive and disjunctive statements.

All this also applies in the third data example, in which a conjunction was tested in combination with two implications. Here, again, the type in Cell 2 2 2 has a different interpretation because it speaks to a different hypothesis than in all prior analyses. Accordingly, the type in Cell 1 1 1 is not interpreted as such. Instead, it is interpreted in the context of three other cells that formally support the hypotheses of conjunction of two implications, but are irrelevant.

Extensions of the present approach will be discussed in future work. For example, temporal dependence can be analyzed using implications. This way, hypotheses can be tested that, in other contexts are tested using Granger causality models (see, e.g., von Eye & Wiedermann, 2015and Bogat et al., 2021). Further, instead of basing hypotheses of implication on Boolean logic, methods that utilize Ternary (three-valued) logic (e.g., Kleene, 1952) can be developed which, in addition to the truth values ‘true’ and ‘false’, includes a third state indicating ‘unknown’ or ‘maybe’ which would allow more fine grained statements about causal directionality.

## Data Availability

The research data in this study are presented in the form of contingency tables.

## Footnotes

1 ^1^ It should be noted that Cell 2 2 2 is the last cell in Tables 3 and 4. The order of “true” and “false” cells is, therefore, inverted in comparison to Tables 5 and 6.
